# Plasma-derived extracellular vesicle surface markers CD45, CD326 and CD56 correlate with the stage of osteoarthritis: a primary study of a novel and promising diagnostic tool of the disease

**DOI:** 10.1038/s41598-023-47074-z

**Published:** 2023-11-16

**Authors:** Jana Matejova, Livia K. Fecskeova, Lucia Slovinska, Denisa Harvanova, Timea Spakova, Jana Bzdilova

**Affiliations:** Associated Tissue Bank, Faculty of Medicine, P. J. Safarik University and L. Pasteur University Hospital in Kosice, Tr. SNP 1, 04011 Kosice, Slovakia

**Keywords:** Biochemistry, Biomarkers, Medical research, Molecular medicine

## Abstract

Recently, there is a growing interest in the research based on extracellular vesicles (EVs) which represent paracrine factors secreted by almost all cell types. Both, normal and pathological cells are able to release various types of EVs with different physiological properties, functions and compositions. EVs play an important role in intercellular communication, mechanism and tissue repair. Moreover, EVs could help not only in the treatment of diseases but also in their diagnostics. This work focused on the evaluation of the potential of EVs being used as biomarkers for the diagnosis of osteoarthritis (OA) based on a comparison of the composition of EVs separated from platelet-poor plasma (PPP) of healthy donors and OA patients at different stages of OA. OA is established as a complex syndrome with extensive impact on multiple tissues within the synovial joint. It is a chronic disease of musculoskeletal system that mainly affects the elderly. Depending on the use of the Kellgren–Lawrence classification system, there are four grades of OA which have a negative impact on patients' quality of life. It is very difficult to detect OA in its early stages, so it is necessary to find a new diagnostic method for its timely detection. PPP samples were prepared from whole blood. PPP-EVs were separated from 3 groups of donors—healthy control, early stage OA, end-stage OA, and their content was compared and correlated. EVs from PPP were separated by size exclusion chromatography and characterized in terms of their size, yield and purity by NTA, western blotting, ELISA and flow cytometry. Detection of surface markers expression in EVs was performed using MACSPlex approach. Inflammatory and growth factors in EVs were analysed using MAGPix technology. Our study confirmed significant differences between EVs surface markers of patients and healthy controls correlating with the age of donor (CD63, CD31 and ROR1) and stage of OA (CD45, CD326 and CD56), respectively. Circulating EVs have been under extensive investigation for their capability to predict OA pathology diagnosis as potential targets for biomarker discovery. Taken together, obtained results indicated that PPP-EVs surface markers could be used as potential biomarkers in the early diagnosis of OA.

## Introduction

Extracellular vesicles (EVs) are membranous structures derived from cells and are considered as important mediators of intercellular communication via transfer of their cargo rich in bioactive proteins, lipids, DNAs and RNAs between cells. The International Society for Extracellular Vesicles (ISEV) defined EVs as “the generic term for particles naturally released from the cell that are delimited by a lipid bilayer and cannot replicate, i.e. do not contain a functional nucleus”^1,2^. The general term EVs is used to define exosomes, microvesicles and apoptotic bodies which differ in their biogenesis, composition and size, although the clear definition of these subgroups varies in the literature. The composition of EVs reflects the physiological and pathological status of the parental cells. The research interest about EVs is exponentially growing mainly because they have been shown to be released by various types of cells or tissues, to be existed in almost all types of body fluids and to be involved in both physiological processes and pathological conditions^3,4^. There is an evidence that EVs are promising candidates for cell-free regenerative medicine^4^ due to their capability to affect cellular phenotype, proliferation and differentiation in a paracrine manner^5^. Moreover, through their heterogeneous composition, EVs are also considered to be a potential source of circulating biomarkers of several diseases. Some studies have suggested increasing EVs level and specific EVs constituents as diagnostic biomarkers in several autoimmune diseases, such as primary Sjögren’s syndrome, systemic lupus erythematosus and systemic sclerosis^6,7^. Burello et al. investigated the surface antigen profile of EVs that could be used in the recognition of transient ischemic attacks^8^. EVs associated RNAs and proteins have been reported as tumor biomarkers for monitoring progression of cancer or its diagnostics^9–12^. Pathological EVs also play an essential task in inflammation and chronic pain diseases^13^. Moreover, the various expression of EVs in the synovial fluid of healthy and osteoarthritic joints proposes EVs as potential diagnostic tool for osteoarthritis (OA)^14^.

OA is the most common form of arthritis, which is reflected by pain of movable joints (wrist of joints, hands and feet, hip joint, knee joint), pain during increased exertion or joint stiffness. The increasing pain negatively affects the quality of patient’s life. It is a disease of the entire joint including structural changes in articular cartilage, subchondral bone, synovial membrane and muscles around the joint^15–17^. Currently, OA is detected mainly by imaging methods or physical examination and there is no appropriate therapy for OA. The Kellgren–Lawrence (KL) grading system of radiographic OA is one of the methods commonly used to estimate the severity of cases^18^. The KL grading scheme was originally described using anteroposterior knee radiographs assigned to grade from 0 to 4^19^. Grades are correlated to increasing severity of OA as follow: *grade 0* (none; signifying no presence of OA), *grade I* (doubtful), *grade II* (minimal), *grade III* (moderate), *grade IV* (severe; signifying severe OA)^19,20^. The most common used treatment of OA is a surgical procedure leading to an artificial joint replacement in the end-stage OA patients. Treatment in the early stages of the disease would be much more suitable for patients and therefore the importance of early diagnosis of OA is underlined and urgently needed.

OA pathogenesis is characterized also by the formation of osteophytes, synovitis, degeneration of menisci and hypertrophy of the joint capsule. Multiple pathophysiological processes are involved in OA progression, including the activation of the innate and immune systems and imbalance between the anabolic and catabolic factors, eventually leading to the degradation and dysfunction of cartilage and bone^15^. As one of the important part of OAs pathophysiology is inflammation, likewise cytokines are estimated as possible candidates for biochemical markers. Cytokines, both pro- and anti-inflammatory, have been studied for their connection with the progression of OA in both human and animal models and their potential use in the diagnosis of OA^21^. Concentration of cytokines were found to be altered depending on the OA stage and on physical activity of patients^22^. Currently, the most important inflammatory mediators in OA pathogenesis are IL-6, IL-1β, IL-15 and TNF-α which are able to activate various signaling pathways that further activate other cytokines and pathological processes^23^.

The composition and amount of EVs in body fluids have been reported to be significantly affected by OA. EVs content may vary in specific types of joint diseases, it is related to disease progression and also contributes to pathological processes. Gao et al. determined that the number of exosomes in the synovial fluid increased from early to severe knee OA without indicated changes in the average particle size^24^. Oba et al. reported that serum of OA patients could contain elevated levels of CD3^+^ CD4^+^ EVs from T-cells^25^. Also, increased levels of annexin V^+^ EVs and annexin V^+^ CD45^−^ CD61^+^ platelet-derived EVs have been reported in the plasma of OA patients compared to healthy controls^26^. Zhao et al. found that the level of EVs isolated from synovial fluid in OA patients was higher compared to healthy controls, but there was no significant difference between early and late-stage OA. They also observed, that expression of lncRNA PCGEM1 in EVs from synovial fluid differ not only between patients with OA and healthy donors, but also between OA stages^27^. Extensive studies suggest that EVs play an essential role in the pathogenesis of OA and their potential use as OA specific biomarkers in the early stages needs to be further investigated.

Blood plasma is one of the abundant sources of EVs and cytokines which can act as an effective tool for the diagnosis of OA by biomarkers. Peripheral blood is easily and routinely collected and does not usually cause excessive discomfort to patients. However, blood plasma contains large amounts of soluble proteins and aggregates which may cause a problem concerning the production of clinical grade EVs in sufficient quantity, high efficiency and without impurities. Between the various techniques available in the meantime, size exclusion chromatography (SEC) has been described as the most efficient method for EVs isolation from biological fluids, which removes major biofluid contaminations, avoids EVs aggregation and retains their functional characteristics^28,29^. The use of commercial columns allows to separate molecules and particles by size under quality controlled and consistent conditions^29^.

The main aim of our work was to evaluate the potential use of EVs as biomarkers for the diagnosis of OA based on comparison of the composition and mainly surface markers expression in samples of EVs separated from platelet-poor plasma (PPP) of healthy donors and OA patients at different stages of OA. EVs were separated using commercial qEV Izon columns and characterized in terms of their size, yield and purity according to ISEV. Furthermore, our work was focused on the evaluation and comparison of the cytokine profiles of PPP and PPP-derived EVs of healthy donors and OA patients. We expect that this work could make a significant progress towards using EVs as potential markers for the early diagnosis of OA.

## Materials and methods

**Figure 1:**
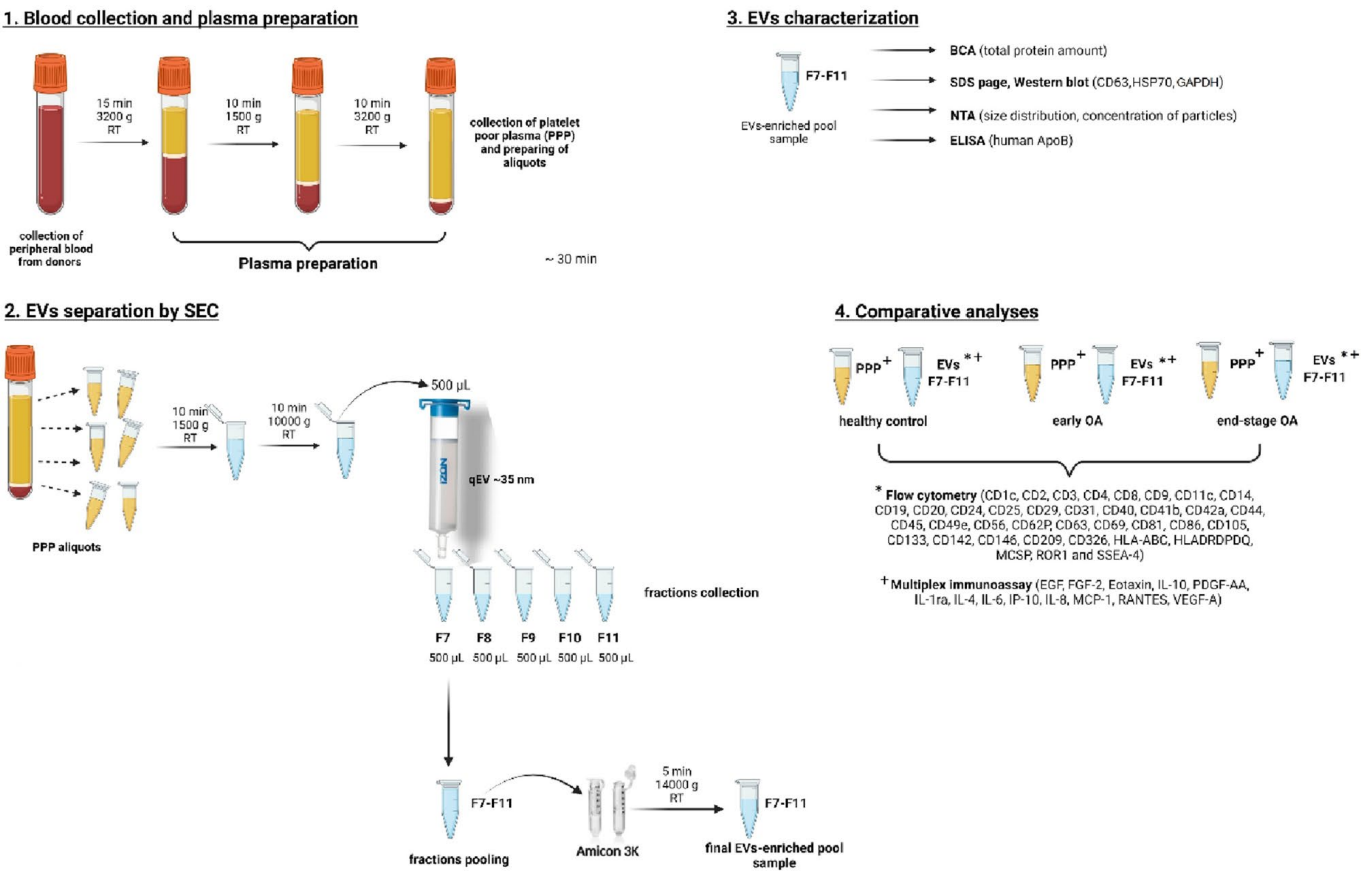
Workflow protocol summarizing: (1) Blood collection and platelet-poor plasma preparation, (2) EVs separation by SEC, (3) EVs characterization and (4) Comparative analyses (created with BioRender.com).

All chemicals and reagents purchased were of reagent grade and used without any further purification. The workflow of our experiments is summarized on Fig. 1.

### Patients and preparation of plasma samples

Collection of the blood sample was in accordance with ethical approval of the P. J. Safarik University Faculty of Medicine and Louis Pasteur University Hospital in Kosice, Slovakia and realized after obtaining informed consent from healthy volunteers and OA patients. Blood was drawn from 9 healthy controls and 21 patients diagnosed with OA, without previous fasting. The healthy controls did not have diagnosed knee OA (KL grade 0). In all patients was confirmed OA radiographically with different grades [early OA with KL grade I–II (n = 11) or end-stage OA with KL grade III–IV (n = 10)]. Participants´ age and sex were recorded (Table 1). Majority of the volunteers were females (60%). Individuals with known acute illness or chronic infectious disease were excluded from the study.

**Table 1:** Participant characteristics.

	Healthy	Early OA	End-stage OA
	n = 9	n = 11	n = 10
Age			
Median	39.00	61.00	63.50
Range	27–47	47–68	42–77
Gender			
Female	9	4	5
Male	0	7	5
BMI ± SD (kg/m^2^)	24.8 ± 2.48	27.8 ± 3.53	32.7 ± 5.88

To prepare PPP samples, 10 mL of whole blood was obtained from each participant into citrate tubes (SARSTEDT AG & Co, Germany) and processed within 35 min of sampling to obtain plasma as it was described previously^30,31^. Briefly, whole blood was firstly centrifuged at 2200×*g* for 15 min at RT, in order to separate the blood into layer with red blood cells, buffy coat rich in white blood cells and platelets in plasma. The buffy coat with the plasma layer were next centrifuged in two steps, 500×*g* for 10 min for leukocyte separation and 2200×*g* for 10 min at RT to obtain the upper part consisting of PPP and the lower part consisting of platelet-rich plasma (PRP)^31^. PPP samples were gently removed to new tubes, aliquoted and stored at − 80 °C. Samples were not exposed to freeze–thaw cycles.

### EVs separation and concentration

EVs from PPP were separated by SEC using qEVoriginal ~ 35 nm columns. Columns were always equilibrated before the experiment, flushed with at least one column volume of 1 × PBS and the whole experiment was performed at RT. First, samples were prepared by centrifugation at 1500×*g* for 10 min to remove any cells and large particles. Next step was the centrifugation of obtained supernatant at 10,000×*g* for 10 min. PPP samples were directly loaded onto the loading frit and fractions were collected immediately in volume 500 µL. Further, 1 × PBS (elution buffer) was loaded and fractions were further collected. 15 fractions were collected during one separation process (1 PPP sample), marked F1–F15. Fractions rich in EVs, F7–F11, were pooled and concentrated 2 times at 14,000×*g* for 5 min using Amicon Ultra 3 K centrifugal filter unit (Merck Life Science, USA). Aliquots were then stored at − 80 °C for subsequent analysis.

### EVs characterization

#### Protein quantification and Western blot analysis

The total protein amount in EVs samples was determined by the colorimetric Pierce™ Rapid Gold bicinchoninic acid (BCA) assay kit (Thermo Fisher Scientific, Waltham, USA), in accordance with the manufacturer’s recommendations and analysed on a multimode reader (TRISTAR, Berthold Technologies). Accordingly, EVs were assayed for Western blot analysis to validate the presence of EVs protein specific markers. Firstly, prepared samples were lysed with 10 × RIPA buffer containing protease/phosphatase inhibitors, 10 min on ice. The global protein content was analysed by SDS-PAGE, followed by Coomassie Blue staining.

Equal protein amount (50 μg) of EVs samples were mixed with non/reducing 4 × Laemmli-buffer in ratio 4:1 and boiled for 5 min at 95 °C. Samples were then separated on 4–10% SDS-PAGE gels and then transferred to PVDF membrane (Bio-Rad Laboratories, California, USA). The membrane was blocked in 5% skimmed milk (Sigma Aldrich, Germany) in TBS-Tween for 1 h at RT. The membrane was then incubated with primary antibodies rabbit anti-CD63 (1:1000), rabbit anti-Hsp70 (1:1000) and mouse anti-GAPDH loading control (1:800), overnight at 4 °C with gently rolling. Secondary antibodies, goat anti-rabbit IgG H&L HRP-conjugated (Abcam, Germany) at 1:20,000 and goat anti-mouse IgG H&L HRP-conjugated at 1:5000 (Invitrogen, Waltham, USA), were used for 1 h incubation at RT with gently rolling. Chemiluminescent signal was generated by the ECL imaging kit (Thermo Fisher Scientific, Waltham, USA) and visualized by X-ray film.

#### Quantification of human ApoB with ELISA

PPP-EVs samples were diluted twofold in Apo ELISA buffer. The concentration (ng/mL) of ApoB100 (specifc for IDLs, LDLs and VLDLs) was measured by designated ELISA (ELISA Pro: Human ApoB: #3715-1HP-2, Mabtech, Sweden) according to the manufacturer’s instructions.

### Nanoparticle tracking analysis

Nanoparticle tracking analysis (NTA) technology (LM10B Nanoparticle Characterization System from Nano Sight, Amesbury, U.K.) was used to carefully analyse the size and concentration distribution of EVs. The samples were diluted in 1 × PBS, mixed well and then injected into the laser chamber. The light scattered by the vesicles is then captured using a video camera. Analysis of the video file allows one to track the motion of each vesicle in two dimensions on a frame-by-frame basis. Video sequences were recorded via a CCD camera operating at 30 frames per second (fps). Five recordings of videos were performed for each sample and analysed by the NanoSight NTA 3.4 Analytical Software Suite. Results were averaged for each sample together and were displayed as a number-weighted particle size distribution.

### Flow cytometry analysis

BD FACSCalibur flow cytometer (Becton Dickinson, Erembodegem, Belgium) equipped with 488 nm and 630 nm lasers was used for EVs measurements by using the MACSPlex Exosome Kit (Miltenyi Biotec, Bergisch Gladbach, Germany). 37 surface APC-conjugated epitopes CD1c, CD2, CD3, CD4, CD8, CD9, CD11c, CD14, CD19, CD20, CD24, CD25, CD29, CD31, CD40, CD41b, CD42a, CD44, CD45, CD49e, CD56, CD62P, CD63, CD69, CD81, CD86, CD105, CD133, CD142, CD146, CD209, CD326, HLA-ABC, HLADRDPDQ, MCSP, ROR1 and SSEA-4 and two isotype controls (mIgG1 and REA control) were measured according to the manufactured instruction. Briefly, 60 µL of all isolated EVs samples were labelled with MACSPlex Exosome Capture Beads overnight in the dark on an orbital shaker (450 rpm) at RT. After incubation, EVs were washed with MACSPlex buffer and centrifuged at 3000×*g* for 5 min. Next, samples were incubated with APC-conjugated detection antibodies for 1 h at RT in dark on an orbital shaker (450 rpm). After washing, the APC signal intensity in each 39 specific bead populations were measured on a Becton Dickinson FACSCalibur using CellQuestPro software (Becton Dickinson). Median fluorescence intensities (MFI) for all the capture beads were corrected for background signal by subtracting the MFI values of each bead obtained from control sample (buffer only) from the MFI values of the respective beads incubated with sample. The measured MFI inside each gate of a separate bead population was normalized to mean tetraspanin CD9/CD63/CD81 MFI values in order to determine the relative levels of a surface marker.

### Multiplex immunoassay

Due to cost consideration for multiplex immunoassay, a group of 23 donors out of the whole 30 donor samples were used. Both PPP and PPP-EVs samples prepared from randomly selected blood samples from each group of volunteers (n = 7 for healthy controls, n = 8 for early OA and n = 8 for end-stage OA patients) were analysed for the presence of cytokines, chemokines and growth factors using 13-plex magnetic bead kit (MILLIPLEX® MAP Kit, Millipore, Merck KGaA, Darmstadt, Germany) according to the manufacturer’s instructions by multiplex immunoassay. The magnetic bead kit contains the following markers EGF, FGF-2, Eotaxin, IL-10, PDGF-AA, IL-1ra, IL-4, IL-6, IP-10, IL-8, MCP-1, RANTES, VEGF-A, which were established by using the MAGPIX Luminex platform. EVs samples were lysed with 10 × RIPA. Each sample was measured in duplicates. Results are presented as MFI of all beads measured for a given analyte and averaged of the two replicates. Concentrations were interpolated to standard curve for each sample and expressed as pg/mL.

### Statistical analysis

GraphPad Prism 6.0 (GraphPad Soft-ware, San Diego, CA, USA) was used for statistical analysis. The distribution of data was tested using the D'Agostino and Pearson omnibus normality test. Normally distributed variables were expressed as mean ± SD and analysed by the One-way analysis of variance test with the post hoc Tukey's Multiple Comparison Test. Non-normally distributed variables were expressed as medians and interquartile range and analysed using the Kruskal–Wallis test with Dunn’s multiple comparison test as post-hoc analysis. The *p* value < 0.05 was considered significant. Pearson’s/Spearman’s rank correlation coefficient (normally/not normally distributed data) was used to examine correlation between biomolecule concentration in plasma and age. A value of *p* < 0.05, *p* < 0.01, *p* < 0.001 was considered as statistically significant, *, **, ***, respectively. The strength of correlation was interpreted via suggestion by Mukaka^32^. Correlation coefficient 0.1–0.3 was considered weak, 0.3–0.5 was considered moderate, and 0.5–1.0 was a strong correlation.

### Unsupervised analysis, PCA

For unsupervised multivariate analysis, principal component analysis (PCA) was performed on the dataset of CD markers of PPP-EVs and on dataset of pro- and anti-inflammatory molecules of PPP and PPP-EVs in 3 studied groups (23 samples; healthy n = 7, early OA n = 8, end-stage OA n = 8) to reduce the dimensionality of the datasets and to reveal the clustering of samples in the study groups. PCA was calculated in R (ver 4.1.1) on scaled variables using the packages FactoMineR^33^, factoextra^34^ and visualized by ggplot2^35^.

### Ethics approval and consent to participate

All subjects gave their informed consent for inclusion before they participated in the study. The study was conducted in accordance with the Declaration of Helsinki, and the protocol was approved by the Ethics Committee of Louis Pasteur University Hospital, Kosice, Slovakia (Approval ID 2020/EK/09066).

## Results

### PPP-EVs separation and characterization

In our work we focused on the characterization of the content of PPP-EVs from 3 groups of donors and their comparison, which could significantly contribute to deeper knowledge in determining OA diagnostic biomarkers. Blood donors (Table 1) were divided into three groups as healthy (KL grade 0), early OA (KL grade I–II) and end-stage OA donors (KL grade III–IV). The obtained EVs were characterized in accordance with MISEV2018^2^. We have submitted all relevant data of our experiments to the EV-TRACK knowledgebase (EV-TRACK ID: EV230032)^36^. Our EV-METRIC is 50%.

**Figure 2:**
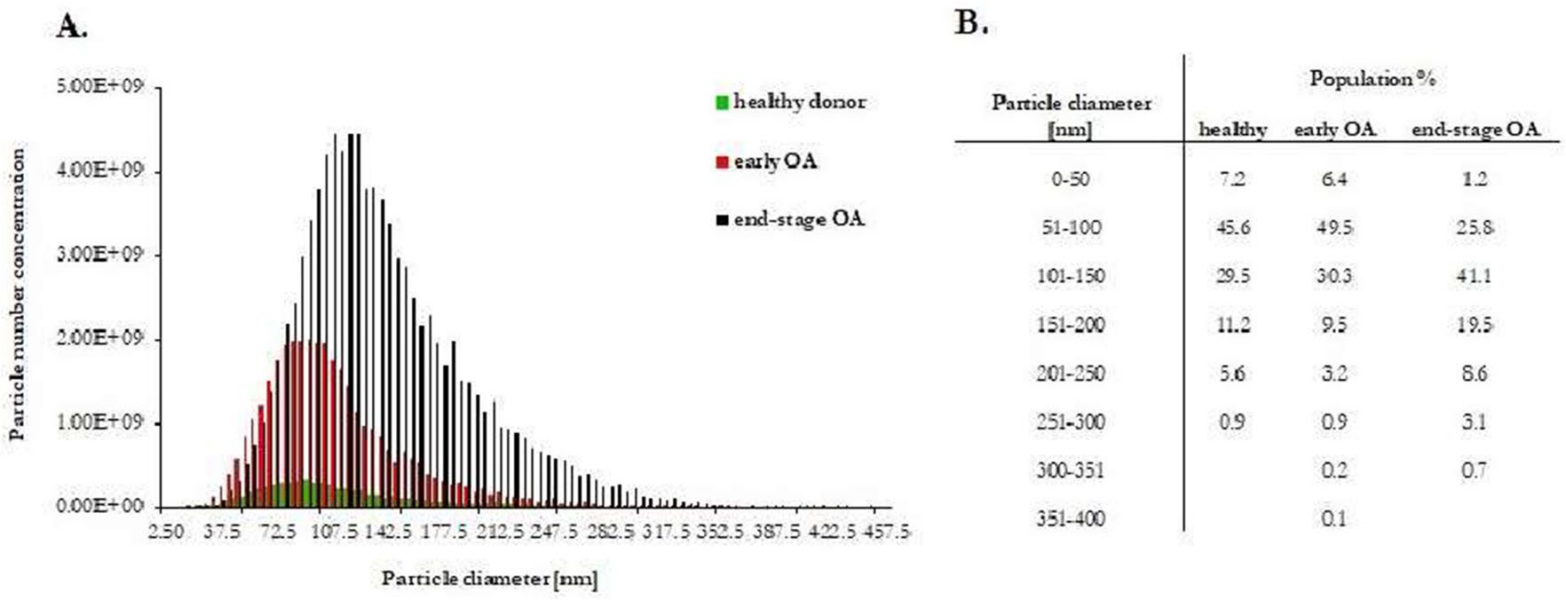
(**A**) Particle concentration and size distribution and (**B**) percentage in various size in pooled fractions F7–F11 assesed by NTA. Results are shown as measurements in one donor of each group (three independent biological samples).

**Figure 3:**
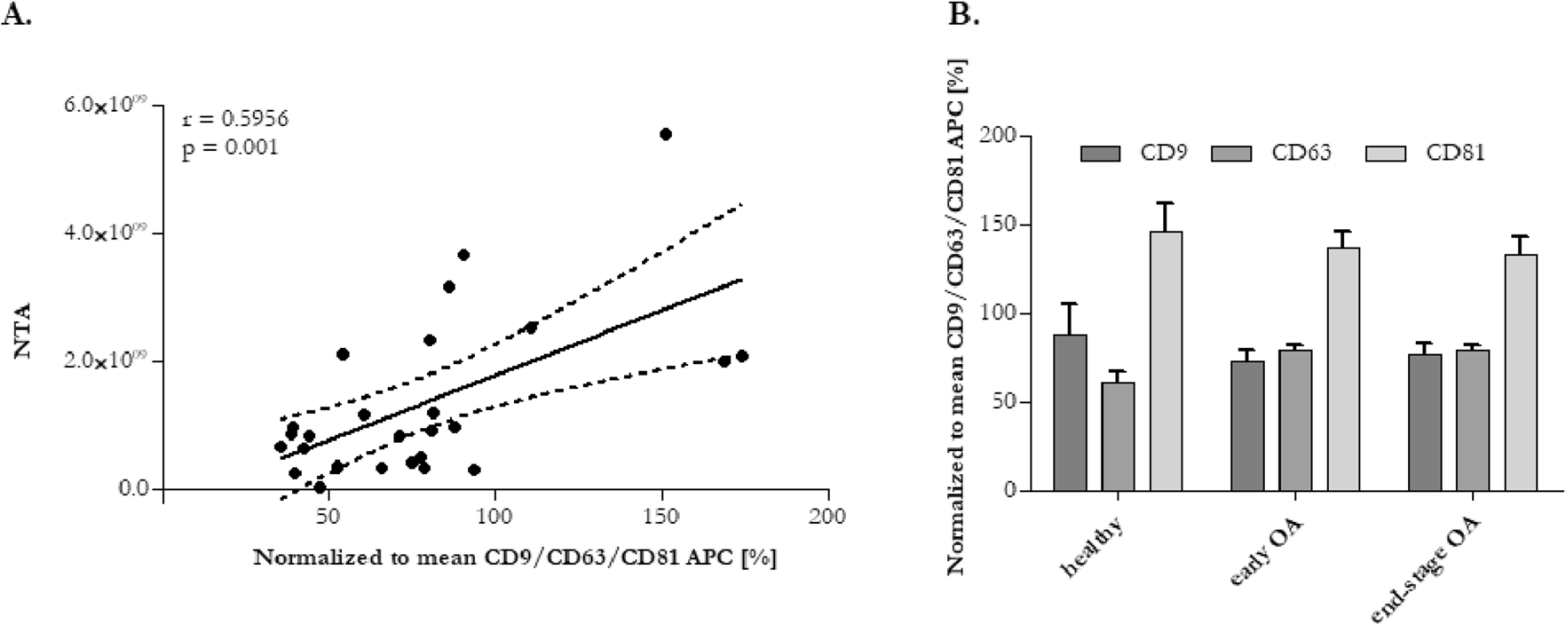
(**A**) Correlation between mean MFI of CD9/CD63/CD81 and N/mL by NTA. *r* = Pearson`s correlation coefficient, *p* < 0.05 was considered significant. (**B**) Background corrected MFI of CD9, CD63, CD81 markers on isolated PPP-EVs.

**Figure 4:**
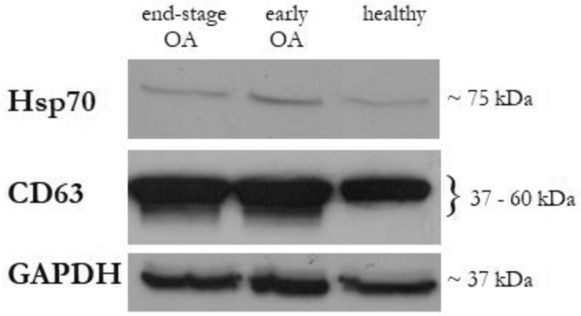
Western blot analysis of CD63 and Hsp70 expression in PPP-EVs from healthy donor, early OA and end-stage OA patients as representative samples. GAPDH was used as loading control.

PPP samples were prepared from the whole blood of each donor. EVs from the PPP were then separated by SEC using qEV columns (Fig. 1). Fractions F7 to F11 were determined as the most EVs enriched fractions. They were pooled and subsequently analysed according to the amount of total protein and particle number and size. Obtained results from every donor in each group were averaged and differences in total protein concentrations and in number and size of EVs are listed in Table 2. Pooled fractions F7–F11 in each donor group contained increasing particle concentrations determined by NTA (Fig. 2A) with size distribution ranging from 50 to 400 nm in diameter. NTA also indicated that the majority of EVs in fractions F7-F11 ranged from 50 to 150 nm in diameter (Fig. 2B) and the concentration of EVs was significantly higher in OA group compared with healthy control (Table 2). We also correlated the MFI normalized to mean CD9/CD63/CD81 (specific markers of EVs) by flow cytometry analysis with number of EVs/mL determined by NTA. Our results showed that mean MFI for CD9/CD63/CD81 strongly correlated with particle concentration obtained by NTA analysis (*r* = 0.5956, *p* = 0.001) (Fig. 3A). Elisa human: ApoB was performed for evaluation of contamination of pooled fractions F7-F11 with non-EV structures/lipoproteins. Our data indicated that PPP-EVs in pooled fractions F7-F11 have less than 0.5% (0.68 ± 0.15 × 10^3^ ng/mL) of contamination marker ApoB-100 of its total amount in PPP samples (178.02 ± 18.6 × 10^3^ ng/mL). In addition, to further confirm the abundance of PPP-EVs in samples, we assessed the expression of the EV-specific tetraspanin surface marker CD63 and the cytosolic heat shock protein Hsp70 by western blotting in pooled fractions F7–F11 from each donor group. The analysis confirmed that EVs markers were positive in all pooled fractions in the identical manner (Fig. 4, Fig. S1).

**Table 2:** Average of total protein concentration (measured by BCA), and particle concentration and size distribution (obtained by NTA) of pooled fractions F7–F11 of healthy (n = 9), early stage OA (n = 11) and end stage OA (n = 10) participant’s.

Donor	Total protein concentration ± SEM (mg/mL)	Number of EVs/mL ± SEM (× 10^10^)	Mean size ± SEM (nm)	Mode size ± SEM (nm)
Healthy (n = 9)	0.80 ± 0.08	0.91 ± 0.2	130.91 ± 8.61	104.33 ± 6.84
Early OA (n = 11)	0.85 ± 0.08	4.02 ± 1.36*	137.06 ± 12.58	110.24 ± 8.57
End-stage OA (n = 10)	1.06 ± 0.17	4.22 ± 1.09*	125.47 ± 7.46	101.65 ± 5.07

Data are expressed as mean ± SEM and *p* < 0.05 (*) was considered statistically significant to control. *p* values were calculated by using a Kruskal–Wallis test.

### Analysis of surface markers of PPP-EVs in donor groups

**Figure 5:**
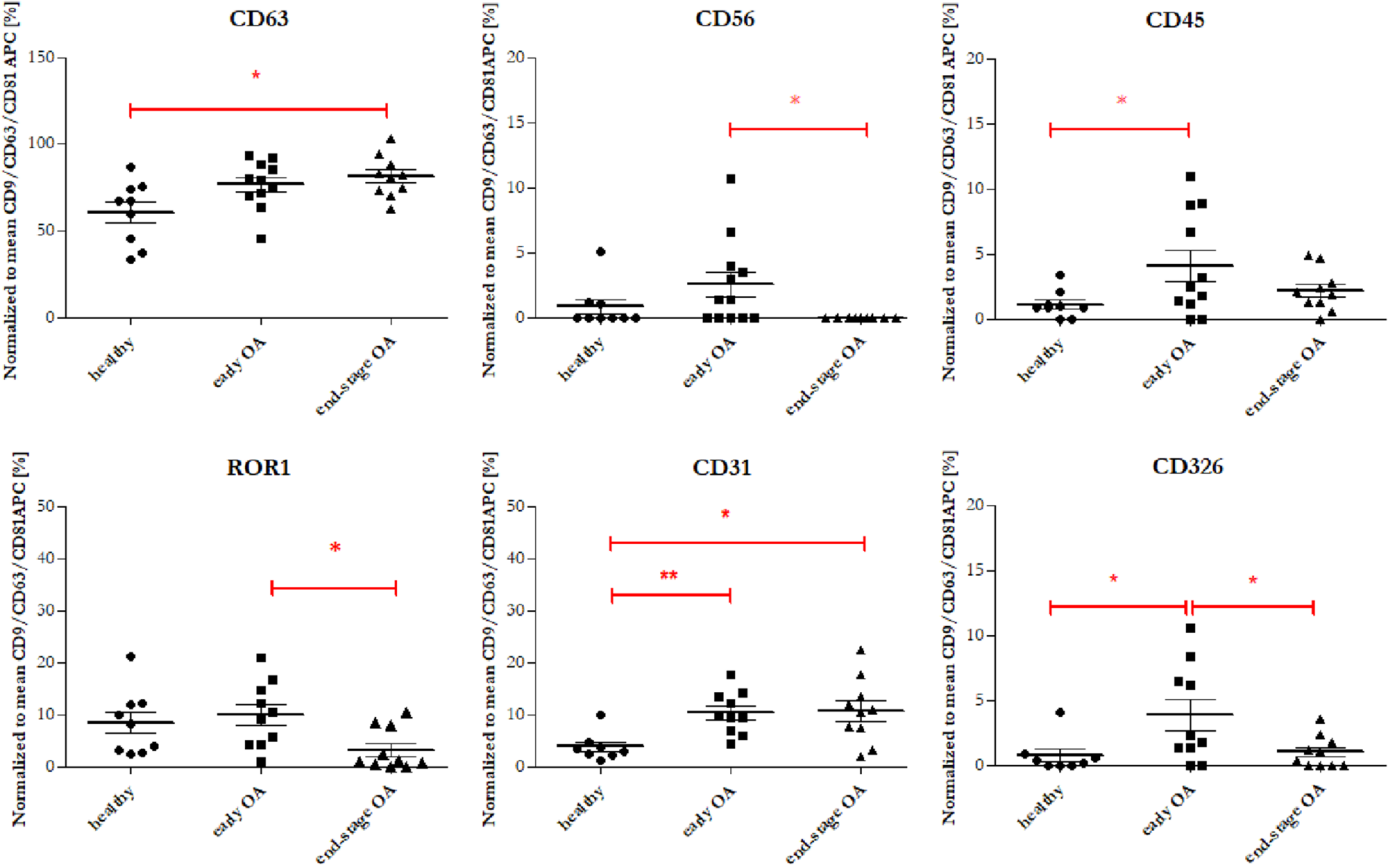
Normalized MFI of the surface protein profiles of the PPP-EVs from healthy blood donors (black filled circle), patients with early OA (black filled square) and patients with end-stage OA (black filled triangle). The data are presented as mean ± SD scatter plots. One-way analysis of variance (ANOVA) with Tukey`s multiple comparison test was applied. ***p < 0.001, **p < 0.01, *p < 0.05 were considered statistically significant.

To characterize PPP-EVs from various donors (healthy vs. OA), we analysed their surface marker profile using a multiplex bead-based platform that allows the detection of 37 surface epitopes simultaneously in one sample. In each experimental group, EVs were positive for the well-established EVs markers CD9, CD63 and CD81. Moreover, expression of CD81 was the highest in each group (Fig. 3B). The MFI of 12 out of 37 measured markers was smaller than the MFI of the isotype controls, suggesting their expression below the detection limit of the assay or even absence in all three study groups. The remaining 25 markers (CD3, CD8, HLA-DRDPDQ, CD56, CD105, CD25, ROR1, CD9, HLA-ABC, CD63, CD40, CD62P, CD81, CD146, CD41b, CD42a, CD24, CD44, CD326, CD133/1, CD29, CD69, CD45, CD31, CD209) exhibited an MFI higher than the corresponding isotype control, demonstrating their presence on the EVs. Significantly different changes were observed in the case of 6 markers (Fig. 5). Expression of CD56, ROR1 and CD326 were significantly higher in the group of patients with early OA compared to the group of patients with end-stage OA. On the other hand, expression of CD326, CD45 and CD31 were significantly higher in the group of patients with early OA compared to healthy control. CD63 and CD31 had significantly higher expression in the group of end-stage OA participants in comparison to healthy control.

**Figure 6:**
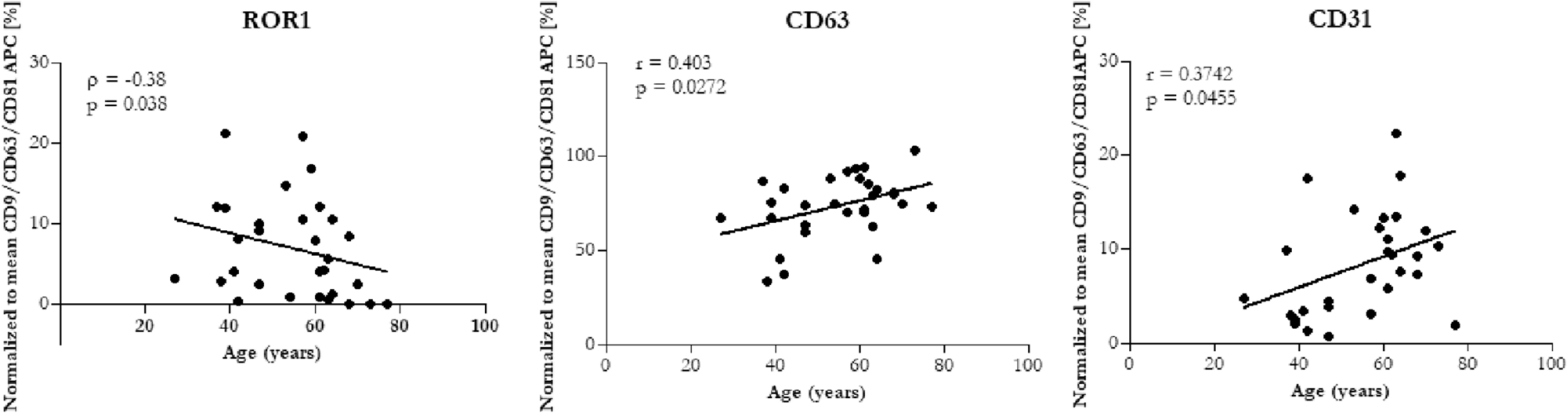
Correlation between age and surface markers of EVs. *r* = Pearson’s coefficient, *ρ* = Spearman’s coefficient; *p* value is based on the Pearson’s/Spearman’s correlation test. *p < 0.05 considered statistically significant.

Because OA is an age-related disorder, we also performed the correlation analysis between the expression of surface markers of EVs and age. Pearson’s (r) or Spearman’s (ρ) rank correlation coefficients between age and measured proteins are shown in Fig. 6. From the 37 cell surface markers only 3 proteins significantly correlated with age. ROR1 was negatively correlated with age of patients (*ρ* = − 0.38, *p* = 0.038), while correlation of CD63 and CD31 with age was positive (*r* = 0.403, *p* = 0.0272; *r* = 0.3742, *p* = 0.0455, respectively). All correlations were considered as moderate.

**Figure 7:**
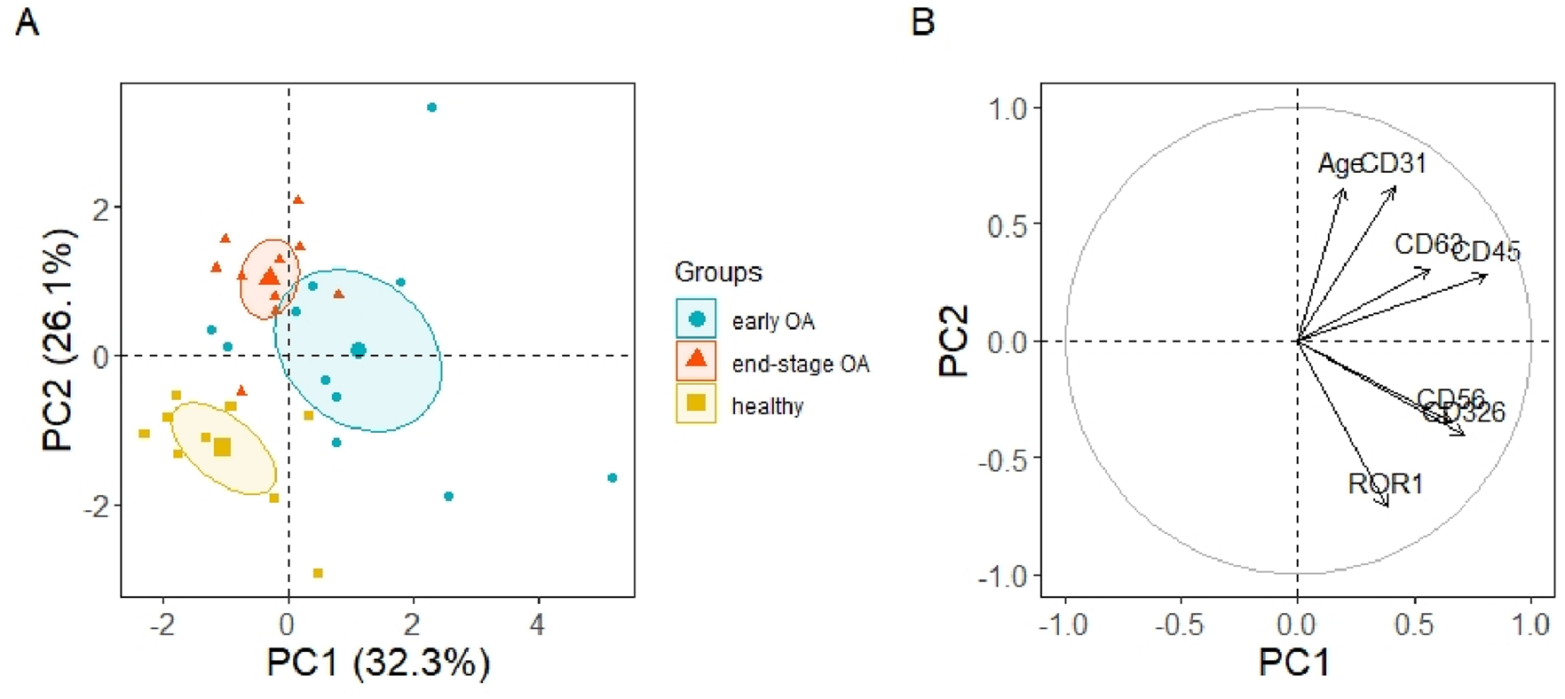
(**A**) PCA individual plot and (**B**) variables plot on the correlation circle of the statistically significant CD markers of PPP-EVs of 30 samples divided into 3 groups (healthy n = 9, early OA n = 11, end-stage OA n = 10). Ellipses represent the 95% confidential interval with centered symbols representing the mean value.

To determine whether the expression of statistically significant CD markers (CD63, CD56, CD45, ROR1, CD31and CD326) of PPP-EVs differed between healthy, early OA and end-stage OA donors in our study, we also evaluated separation between experimental groups using unsupervised PCA (Fig. 7). PCA explained 58.4% of the data distribution in PPP-EVs samples (PC1 32.3%, PC2 26.1%) and resulted in a clear separation of the healthy group from the two OA groups (Fig. 7A). This separation is mainly along the PC2 axis and is correlated with age, CD31 and ROR1, but also along the PC1 axis in correlation with CD45, CD56, CD326 and CD63. Surface markers CD56 and CD326 mainly contribute to the mild separation between the early and end-stage OA clusters, whilst CD63 and CD45 contribute to the separation between healthy and early-stage OA (Fig. 7B).

### Analysis of the expression profiles of cytokines and chemokines in PPP and PPP-derived EVs of donor groups

**Figure 8:**
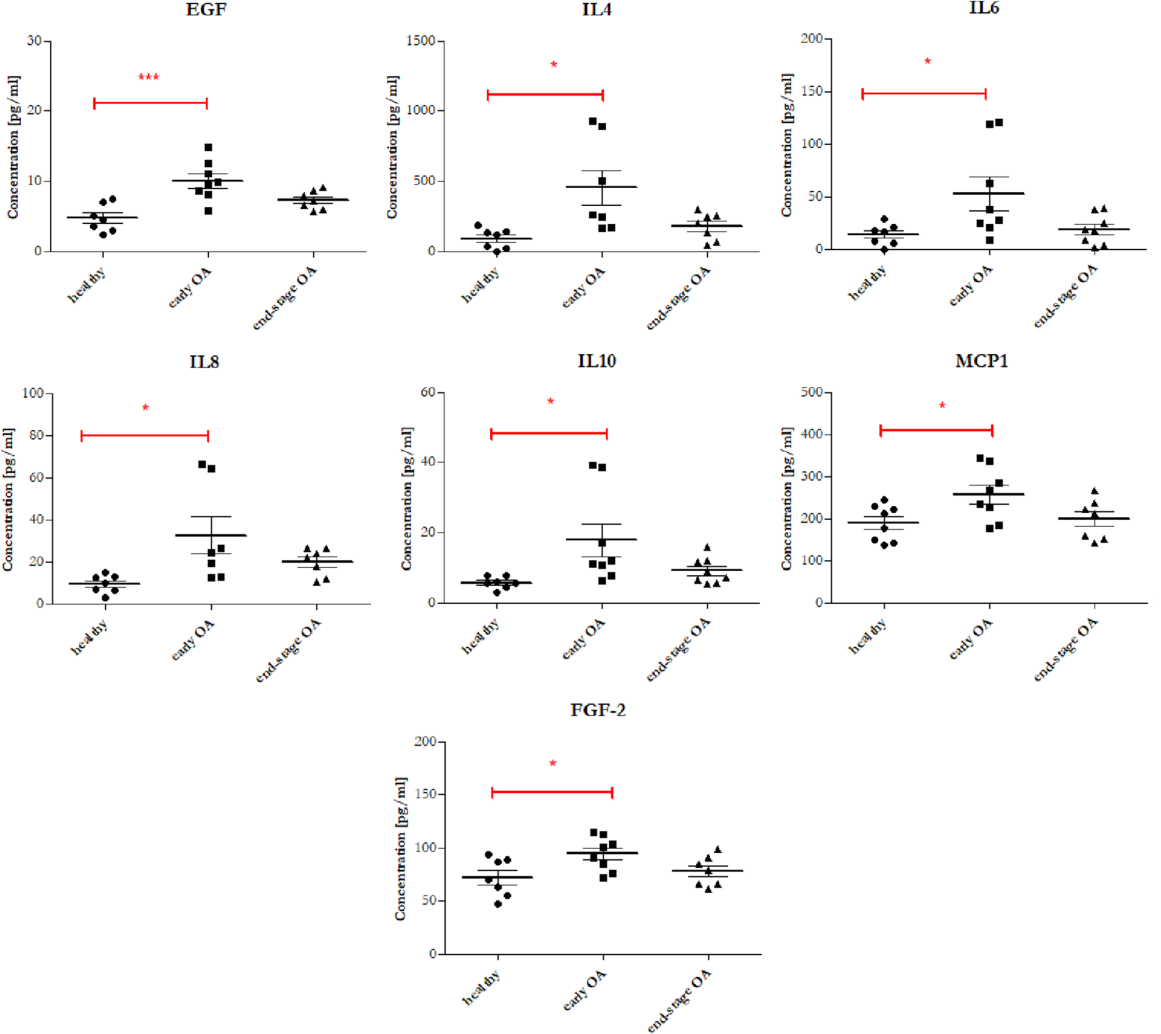
Concentration of biomolecules measured in PPP from healthy blood donors (black filled circle), patients with early stage of OA (black filled square) and patients with end-stage of OA (black filled triangle). The data are presented as mean ± SD scatter plots. One-way analysis of variance (ANOVA) with Tukey’s multiple comparison test was applied. ***p < 0.001, **p < 0.01, *p < 0.05 were considered statistically significant.

**Figure 9:**
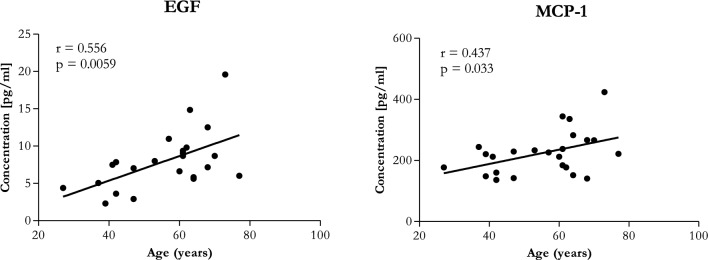
Correlation between age and concentration of studied biomolecules in PPP, *r* = Pearson’s coefficient, *p* value is based on the Pearson’s correlation test. *p < 0.05 and **p < 0.01 considered statistically significant.

In order to monitor the severity of the OA disease, changes in the concentration of biomolecules in PPP and PPP-derived EVs between all studied groups were evaluated and compared in this study. Cytokines (IL-10, IL-1RA, IL-4, IL-6, IL-8), chemokines (eotaxin, IP-10, MCP-1, RANTES) and growth factors (EGF, FGF-2, PDGF, VEGF) were analysed in each donor group. In the case of PPP-derived EVs there were no significant differences between the studied groups and concentrations of biomolecules were very low (Table 3B). In the case of IL-1RA, eotaxin, IP-10, RANTES, PDGF, and VEGF in PPP there were no significant differences in concentration according to the grade of OA (Table 3A). On the other hand, concentrations of IL-10, IL-4, IL-6, IL-8, EGF, FGF-2, and MCP-1 were significantly higher in the group of patients with early OA compared to the healthy control (Table 3A, Fig. 8). Next, the relationship between plasma cytokines and age was assessed. As show in Fig. 9, growth factor EGF and chemokine MCP-1 were positively correlated with age (*r* = 0.556, *p* = 0.0059 and *r* = 0.437, *p* = 0.033, respectively). Correlation of EGF with age was strong and correlation of MCP-1 with age was moderate.

**Table 3:** The expression profiles of cytokines in the PPP (A.) and PPP-derived EVs (B.) between donor groups.

A	Marker (pg/mL)	Mean ± SD (healthy)	Mean ± SD (early OA)	Mean ± SD (end-stage OA)	*p* value	Assay sensitivity (pg/mL)^#^
	EGF	4.68 ± 1.98	10.00 ± 2.79	7.28 ± 1.31	0.0011*	2.8
	FGF-2	72.2 ± 18.17	94.36 ± 15.95	77.97 ± 14.14	0.0397*	7.6
	Eotaxin	102.9 ± 43.84	147.7 ± 47.37	121.2 ± 24.43	0.1227	4.0
	IL-10	5.744 ± 1.67	17.81 ± 13.41	9.116 ± 3.703	0.0276*	1.1
	IL-1RA	30.98 ± 15.02	28.56 ± 7.583	30.82 ± 11.09	0.9055	8.3
	IL-4	90.88 ± 71.17	451.8 ± 333.3	176.1 ± 98.87	0.0105*	4.5
	IL-6	13.97 ± 10.05	52.68 ± 44.23	18.83 ± 14.21	0.0263*	0.9
	IL-8	9.425 ± 4.269	32.37 ± 23.22	19.82 ± 6.671	0.0243*	0.4
	IP-10	429.4 ± 121.4	478.0 ± 258.1	418.8 ± 185.0	0.8216	8.6
	MCP-1	188.9 ± 43.09	256.3 ± 62.97	198.6 ± 48.03	0.0385*	1.9
	PDGF	125.6 ± 38.52	111.1 ± 47.75	140.5 ± 41.48	0.4517	0.4
	RANTES	637.2 ± 376.9	610.7 ± 341.4	493.1 ± 306.1	0.5109	1.2
	VEGF	34.4 ± 38.81	78.24 ± 55.23	25.05 ± 37.33	0.0731	26.3

B	Marker (pg/mL)	Median (IQR) (healthy)	Median (IQR) (early OA)	Median (IQR) (end-stage OA)	*p* value	Assay sensitivity (pg/mL)^#^
	EGF	3.2 (2.095)	3 (0.675)	2.775 (0.722)	0.9129	2.8
	FGF-2	12.99 (7.03)	11.35 (4.89)	13.53 (6.36)	0.8415	7.6
	Eotaxin	3.62 (1.195)	3.62 (0.775)	3.26 (2.737)	0.8336	4.0
	IL-10	0.67 (0.35)	0.67 (0.663)	0.65 (1.908)	0.8799	1.1
	IL-1RA	4.04 (5.375)	4.04 (2.545)	3.64 (1.805)	0.968	8.3
	IL-4	2.488 (4.485)	4.73 (3.695)	0 (3.548)	0.1556	4.5
	IL-6	0.21 (1.85)	0.105 (1.33)	0.105 (1.57)	0.9353	0.9
	IL-8	0.7 (1.048)	0.7 (0.7525)	0.57 (0.63)	0.8531	0.4
	IP-10	11.14 (4.8)	8.603 (4.071)	9.52 (2.777)	0.1133	8.6
	MCP-1	2.8 (0.982)	2.8 (0.817)	2.64 (0.74)	0.8132	1.9
	PDGF	0.915 (0.898)	0.82 (0.465)	1 (1.21)	0.99	0.4
	RANTES	13.92 (20.47)	14.76 (30.84)	28.83 (23.66)	0.1703	1.2
	VEGF	0 (16.04)	2.788 (11.35)	0 (0)	0.358	26.3

Values are expressed as mean ± SD or median and interquartile ranges (IQR). *p* values were calculated by using a Kruskal–Wallis test or One-way analysis of variance.

*p < 0.05 was considered statistically significant.

^**#**^Minimal detectable concentration.

**Figure 10:**
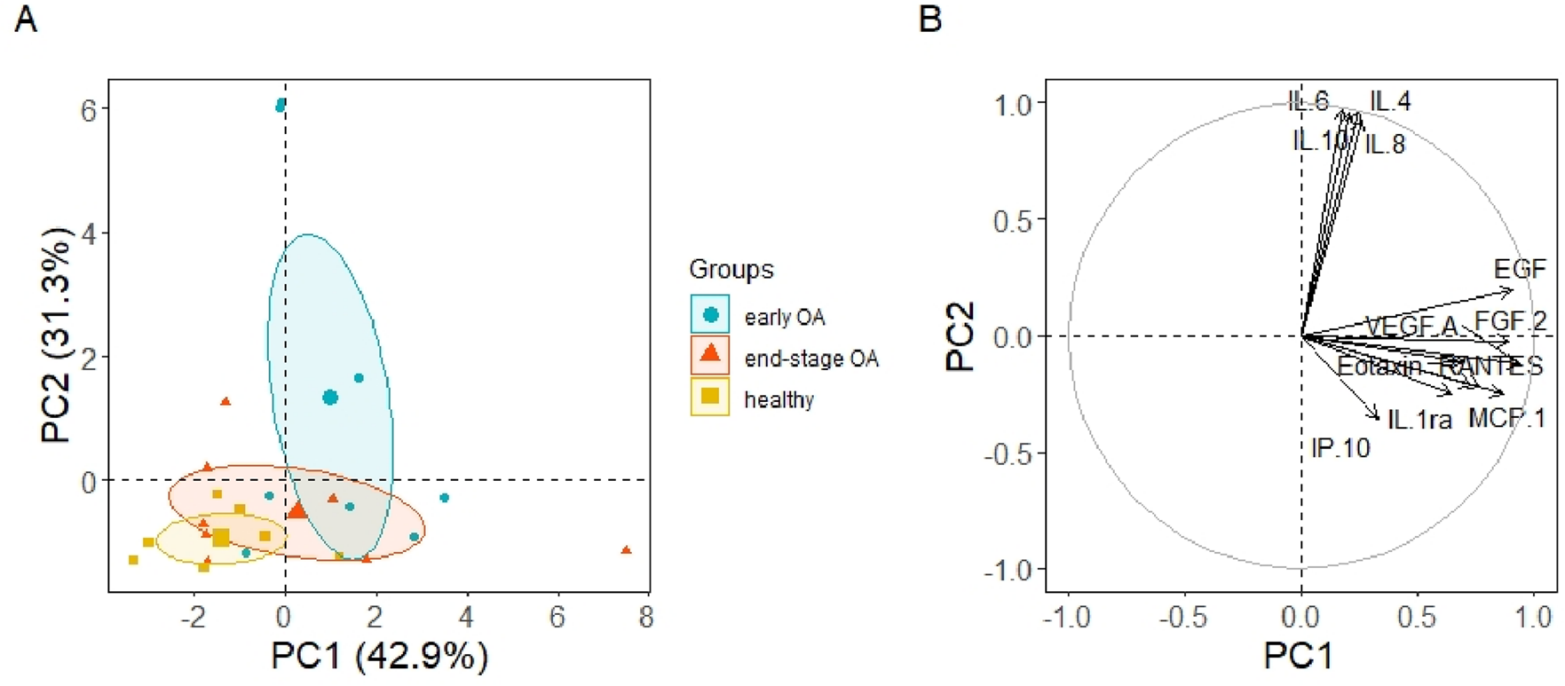
(**A**) PCA individual plot and (**B**) variables plot on the correlation circle of inflammatory markers (cytokines, chemokines and growth factors) in PPP of 23 samples, divided into 3 groups (healthy n = 7, early OA n = 8, end-stage OA n = 8). Ellipses represent the 95% confidential interval with centered symbols representing the mean value.

Further PCA of inflammatory markers in PPP explained 74.2% of the data distribution (PC1 42.9%, PC2 31.3%, Fig. 10) and resulted in three clusters. While the cluster of the end-stage OA overlapped with the healthy cluster, the early stage OA cluster clearly separated from those. This separation was mainly correlated with the cytokines IL-6, IL-8, IL-4, IL-10 (along the P2 axis) (Fig. 10B), supporting that inflammatory cytokines are specific for the early stage of OA and may be indicative of that, similarly to Fig. 8.

## Discussion

EVs are small lipid membrane vesicles released by almost all kinds of cells into the extracellular space and thus have great potential to indicate various changes in physiological and pathological processes occurring in human body. EVs can be easily sampled from peripheral blood and thus they are attractive targets for prognostic testing based on disease biomarkers^37^. Nowadays, there is still no drug for the treatment of OA, patients are either treated symptomatically or in the end-stage of the disease endoprosthesis is done on severely damaged joints. For this reason, there is a high interest in trying to find biomarkers to diagnose OA in the early stages and thus improve the quality of life of affected patients by slowing down the progression of OA. Recently, more and more evidences show that EVs play very important role not only in the progression and treatment of OA, but also in its diagnosis^38^. In this study, we provide evaluation of the potential use of EVs as biomarkers for the diagnosis of OA based on comparison of the composition of EVs separated from PPP of three groups of blood donors (healthy, early OA and end-stage OA donors). A total of 21 OA cases and 9 healthy controls were included. To the best of our knowledge, this is the first report describing the isolation and surface marker detection of EVs from PPP of OA patients.

SEC is considered as a suitable method for EVs separation, which can be easily adapted to many research and clinical laboratories^29^. SEC is in comparison with other methods low cost, less time consuming and requires only small amount of starting material^39^. Generally, SEC separates molecules by differences in their size as they pass through a resin packed in a column. Compared to other separation methods, SEC alters minimally EVs profiles, results in the purest EVs fractions and is able to remove most of the circulating proteins contained in a body fluid^28,30,40^. In this work, PPP samples were prepared from the whole blood of each donor and EVs were then separated by SEC using qEV columns. Altogether, we separated and analysed high number of PPP-EVs from 9 healthy donors, 11 donors with early stage of OA and 10 donors with end stage of OA. The yield of EVs from our PPP samples was in a similar range as yield of EVs from plasma samples from other studies^41,42^. First of all, we demonstrated that PPP-EVs concentration was higher in patients with early OA and end-stage OA compared to healthy donors, but no differences in size were found between groups.

It has been reported, that EVs in blood plasma could be co-isolate with other particles, including lipoproteins. Therefore, obtained higher yield of EVs from SEC may not completely reflect only a large number of EVs. NTA also cannot differentiate between EVs and lipoproteins. To address this issue, we performed Elisa human: ApoB. Apolipoprotein ApoB is the best negative marker for evaluation of contamination with non-EV structures/lipoproteins by working with plasma. ApoB-100 standard was supplied as purified low-density lipoprotein (LDL). Gaspar et al. in their protocol detected ApoB-100 in increasing manner from 0.16 to 23.77% in individual fractions F7 to F10^29^. Our data showed that PPP-EVs pooled fractions F7-F11 have less than 0.5% of contamination marker ApoB-100 of its total amount in PPP samples. The obtained results suggested that separation by SEC can efficiently enrich EV particles and remove highly abundant contaminant proteins from the plasma. Whereas NTA is not specific for EVs characterization, because it is unable to determine the phenotype of the EVs, we correlated the MFI normalized to mean CD9/CD63/CD81 by flow cytometry analysis with number of EVs/mL determined by NTA. The strong positive correlation between mean MFI for CD9/CD63/CD81 and concentration obtained by NTA analysis is in agreement with the results of Vacchi et al.^43^.

After confirming the characteristics of separated PPP-EVs (according to ISEV^2^), we further analysed theirs surface markers in each donor group. EVs surface proteins can potentially reflect the cellular origin and molecular pathology in different diseases and therefore analysis of EVs surface markers belong predominantly to the main characteristics of EVs^44–46^. However, to the best of our knowledge, there is a lack of studies which evaluated the surface markers of PPP-EVs in OA donors in case of their potential purpose as biomarkers for the diagnosis of OA. Surface marker profile of PPP-EVs from various donor group (healthy vs. OA) was analysed by a multiplex bead-based platform (simultaneous detection of 37 surface epitopes in one sample). The analysed proteins were normalized against the CD9/CD63/CD81 signal to minimize variation between different patients. We aimed to investigate whether the composition of EVs in the plasma of OA patients is different as compared to healthy controls and whether OA-specific EVs can be detected. In this work were found significant differences between healthy and OA donors in the case of 6 markers, namely CD56, ROR1, CD326, CD45, CD63 and CD31. Even though markers ROR1, CD31 and CD63 show statistically significant difference between healthy and early- and/or end-stage OA, based on correlations (Fig. 5) and PCA analysis (Fig. 7), these markers also positively correlate with the age of patients. It is likely that the different expressions (especially in the case of ROR1 and CD31) are reflecting the different age groups of our donor groups (Table 1), and only partially relate to OA or not at all. These markers are probably associated with age rather than with the stage of OA, since age increased with disease progression. Important surface markers whose expression in PPP-EVs were highest in patients with early stage OA were CD56, CD45 and CD326. However, based on PCA analysis, CD63 is less likely to be affected by age (Fig. 7), and together with CD45 represent markers contributing to the separation between healthy and OA patients along the PC1 axis. Based on our results, the role of CD63 in the OA is confusing and it should be more examined in the future. On the other hand, the strong correlation of CD326 with CD56 towards the separation between early- and end-stage OA (Fig. 7) indicate that these markers could serve for the differentiation between the stages of OA (early vs. end-stage). These changes in expression of CD45, CD56 and CD326 could indicate their potential in diagnosing the severity of OA and could be potentially use as biomarkers for the OA diagnosis.

Infiltrating immune cells play a central role in degenerative joint disease associated with OA. Natural killer (NK) cells are main tissue-infiltrating lymphocyte subset in patients with OA and can be detected in inflamed synovial tissue at an early stage of the disease. The expression of CD56 (neural cell adhesion molecule, a natural killer cell marker) is most associated with NK cells^47^. CD45 (lymphocyte common antigen) is a protein tyrosine phosphatase that is specifically expressed in leucocytes^48^. Huss et al. observed that CD45^+^ CD56^+^ CD3^−^ NK cells are a principal leukocyte infiltrate in synovial tissue from OA patients undergoing primary and revision total joint replacement^49^. An interesting fact is, that in our study the highest expression of CD56 and CD45 was detected in PPP-EVs of patients with early OA.

Up to date, the pathogenesis of OA is not completely understood, but it is generally known, that both pro-inflammatory and anti-inflammatory cytokines has a pivotal role in the onset, development and progression of OA^22^ and thus they could be considered also as possible way for OA diagnosis. Concentrations of cytokines and chemokines were found to be varied depending on the OA stage and activity^50–53^. Silvestri et al. detected increased concentration of serum soluble IL-4 receptor in all OA patients in comparison with healthy control individuals^54^. Wang et al. showed that IL-18 in plasma, synovial fluid and articular cartilage obtained from knee OA patients was significantly higher compared to healthy controls^52^. Accordingly, IL-1β, IL-6 and TNF-α in synovial fluid were significantly higher in the early stage than in the end-stage of knee OA and correlated with pain^55^. Koh et al. indicated that plasma IL-8 and synovial fluid IL-18 may be associated with the pathogenesis of OA^56^. Barker et al. observed decreased levels in both serum IL-10 and IL10/TNFα ratio in end-stage OA patients compared to moderate OA what could indicate its prognostic value^53^. In our work were also analysed the expression profiles of cytokines (IL-10, IL-1RA, IL-4, IL-6, IL-8), chemokines (eotaxin, IP-10, MCP-1, RANTES) and growth factors (EGF, FGF-2, PDGF, VEGF) in PPP and PPP-derived EVs from three donor groups. However, no significant differences between the studied groups were detected in PPP-EVs. In the case of PPP, no significant changes in the level of IL-1RA, eotaxin, IP-10, RANTES, PDGF and VEGF between end-stage OA and early stage OA or healthy controls were observed. It is important to mention, that concentrations of IL-10, IL-4, IL-6, IL-8, EGF, FGF-2, and MCP-1 were significantly higher only in the group of patients with early OA compared to the healthy control but the concentration of these biomarkers in end-stage OA were similar to those in the healthy control, indicating that inflammation may be better recorded in the early stage. In addition to the fact that significant changes in the concentration of EGF and MCP-1 were observed between healthy and early OA, these markers were also positively correlated with age. Recent studies also showed that MCP-1 positively correlated with age^57^. Moreover, the PCA clustering of the end-stage OA patients together with healthy donors indicate that the inflammatory profile of PPP in the late stage OA is similar to those in healthy individuals and at this stage can no longer serve as biomarkers of end-stage OA.

## Conclusions

As potential targets for biomarker discovery, circulating EVs have been extensively under investigation for their capability to predict OA pathology diagnosis. Nowadays, it is difficult to reach the proper data on OA biomarkers because of heterogeneity not only in reporting data of various studies, but also of variability and complex biochemical and physical composition of biological samples. Therefore, separation of EVs from body fluids for downstream analysis is still questionable. In this study, SEC method was successfully used for EVs separation from PPP, resulting in the optimum EVs yield and high purity. PPP-EVs were separated from 3 groups of donors (healthy, early OA, end-stage OA patients, respectively) and their content was compared and correlated.

Our pilot study confirmed that PPP-EVs were positive to typical EVs surface markers CD9, CD63 and CD81 in each donor group, with the highest expression of CD81. There were found significant differences between *EVs surface markers* of patients and healthy controls: CD56, ROR1 and CD326 were significantly higher in patients with early OA compared to the end-stage OA group; CD326, CD45 and CD31 were significantly higher in patients with early OA compared to healthy control; CD63 and CD31 were significantly higher in end-stage OA group compared to healthy control. We further analysed our data based on age of patients and concluded that CD63, CD31 and ROR1 surface markers of EVs were positively correlated with the age of donor. On the other hand, CD45, CD326 and CD56 were correlated with the stage of OA. Next, we examined the profiling of selected *OA-related biomolecules* in PPP and PPP-EVs in OA/healthy donors. No significant differences were found between the studied groups in PPP-EVs samples. Even though we identified differences in *cytokines* between OA patients and healthy individuals in PPP: concentrations of cytokines IL-6, IL-8, IL-4, IL-10 were significantly higher in patients with early OA compared to the healthy control; moreover, growth factor EGF and chemokine MCP-1 were positively correlated with age.

*In summary*, obtained data suggest, that two EVs surface markers—CD31 and ROR1 correlating with age of donors and three EVs surface markers—CD45, CD326 and CD56 correlating with the early stage of OA, could have the potential of being utilized as biomarkers and may have a bright future in OA diagnosis. Moreover, our results could provide a new way to identify specific OA biomarkers using EVs for a more effective therapeutic strategy.

### Supplementary Information

Supplementary Figure S1.

## Data Availability

The datasets used and/or analysed during the current study are available from the corresponding author on reasonable request.

## Abbreviations

EVs: Extracellular vesicles
ISEV: The International Society for Extracellular Vesicles
MISEV: Minimal information for studies of extracellular vesicles
KL: The Kellgren–Lawrence grade
OA: Osteoarthritis
PCA: Principal component analysis
PPP: Platelet-poor plasma
PRP: Platelet-rich plasma
SEC: Size exclusion chromatography
NK: Natural killer cells
