# Supplementary material for: Plasma-derived extracellular vesicle surface markers CD45, CD326 and CD56 correlate with the stage of osteoarthritis: a primary study of a novel and promising diagnostic tool of the disease
Source: Sci Rep. 2023 Nov 16;13:20071. doi: 10.1038/s41598-023-47074-z (PMC10654566; doi:10.1038/s41598-023-47074-z)
Supplement: Supplementary file 1 — Supplementary Figure S1. [file 41598_2023_47074_MOESM1_ESM.docx]

**Supplementary information**


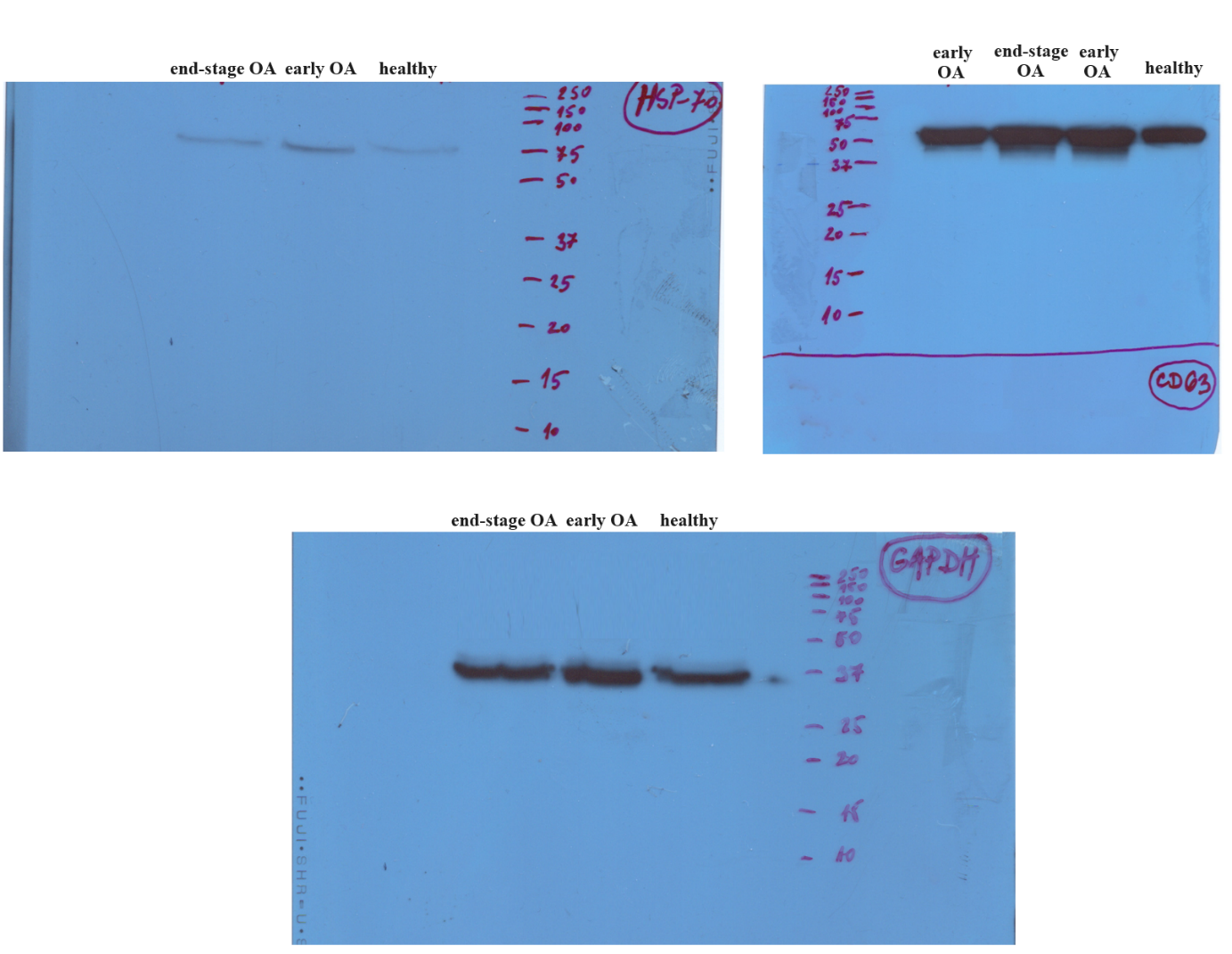


**Supplementary Figure S1 Scans of original western blots visualized on X-ray films.** Western blot analysis of CD63 and Hsp70 expression in PPP-EVs from healthy donor, early OA and end-stage OA patients as representative samples. GAPDH was used as loading control.
